# Dendritic cells transfected with DNA constructs encoding CCR9, IL-10, and type II collagen demonstrate induction of immunological tolerance in an arthritis model

**DOI:** 10.3389/fimmu.2024.1447897

**Published:** 2024-08-05

**Authors:** Marina S. Fisher, Vasily V. Kurilin, Aleksey S. Bulygin, Julia A. Shevchenko, Julia G. Philippova, Oleg S. Taranov, Elena K. Ivleva, Amir Z. Maksyutov, Sergey V. Sennikov

**Affiliations:** ^1^ Laboratory of Molecular Immunology, Federal State Budgetary Scientific Institution Research Institute of Fundamental and Clinical Immunology, Novosibirsk, Russia; ^2^ Department of Microscopic Research, State Research Centre for Virology and Biotechnology «Vektor», Koltsovo, Russia; ^3^ Theoretical Department, State Research Center for Virology and Biotechnology “Vektor”, Koltsovo, Russia

**Keywords:** immunological tolerance, tolerant dendritic cells, experimental arthritis, antigen-specific dendritic cells, DNA-constructs, T-regulatory cells

## Abstract

**Introduction:**

Restoring immune tolerance is a promising area of therapy for autoimmune diseases. One method that helps restore immunological tolerance is the approach using tolerogenic dendritic cells (tolDCs). In our study, we analyzed the effectiveness of using dendritic cells transfected with DNA constructs encoding IL-10, type II collagen, and CCR9 to induce immune tolerance in an experimental model of arthritis.

**Methods:**

Dendritic cell cultures were obtained from bone marrow cells of Balb/c mice. Dendritic cells (DCs) cultures were transfected with pmaxCCR9, pmaxIL-10, and pmaxCollagen type II by electroporation. The phenotype and functions of DCs were studied using enzyme-linked immunosorbent assay (ELISA) and flow cytometry. Migration of electroporated DCs was assessed *in vitro*. Induction of antigen-collagen induced arthritis (ACIA) was carried out according to the protocol in Balb/c mice. DCs were then administered to ACIA mice. The development of arthritis was monitored by measuring paw swelling with a caliper at different time points. The immunological changes were assessed by analyzing the content of antibodies to type II collagen using enzyme immunoassay. Additionally, a histological examination of the joint tissue was conducted, followed by data analysis.

**The results are as follows:**

DCs were obtained, characterized by reduced expression of CD80, CD86, and H-2Db (MHC class I), increased expression of CCR9, as well as producing IL-10 and having migratory activity to thymus cells. Transfected DCs induced T-regulatory cells (T-reg) and increased the intracellular content of IL-10 and TGF-β in CD4^+^T cells in their co-culture, and also suppressed their proliferative activity in response to antigen. The administration of tolDCs transfected with DNA constructs encoding type II collagen, IL-10, and CCR9 to mice with ACIA demonstrated a reduction in paw swelling, a reduction in the level of antibodies to type II collagen, and a regression of histological changes.

**Conclusion:**

The study presents an approach by which DCs transfected with DNA constructs encoding epitopes of type II collagen, IL-10 and CCR9 promote the development of antigen-specific tolerance, control inflammation and reduce the severity of experimental arthritis through the studied mechanisms: induction of T-reg, IL-10, TGF-β.

## Introduction

Some of the main characteristics of autoimmune diseases (AIDs) are loss of tolerance and induction of immune response to the body’s own antigens, resulting in damage to cells and tissues of the body (1). Therefore, restoration of immune tolerance remains one of the main directions of therapy, which aims to eliminate the causes of diseases. Currently, approved methods for AID treatment include continuous administration of immunosuppressive drugs, targeted biological drugs, and synthetic inhibitors of signaling pathways. These drug groups have nonspecific immunosuppressive effects, including drugs with targeted action that block specific inflammatory agents and affect them in general, which leads to certain side effects (2). The used drugs can cause immunosuppression, leading to the risk of chronic infections or cancer (2–4). Modern AID treatment options also rarely lead to a cure or drug-free remission (5, 6). Thus, the development of new methods of AID therapy that lead to persistent remission and minimize side effects remains a relevant scientific task.

One of the methods that contribute to the restoration of immunologic tolerance is the actively developing approach that involves tolDCs. It is known that tolDCs have the ability to trigger and support both central and peripheral tolerance (7). The use of tolDCs was successful in some clinical trials (8–10). Still, there are some drawbacks that need to be taken into account to improve the development of effective tolDCs therapy. Nonspecific tolDCs have the potential to suppress the functional activity not only of autoreactive T cells but also of other immune cells. This again brings one back to the risk of the mentioned side effects: infectious and oncologic diseases, nephro- and hepatotoxicity. In the production of antigen-specific DCs, the problem of selecting antigens for loading arises, since several autoantigens are involved in the pathogenesis of many AIDs, and tolerance to only one of them may not lead to the expected clinical effect. A possible solution to the problem of selecting antigens for therapy would be the simultaneous use of multiple autoantigens, peptides, or transfection of exogenous DNA encoding multiple immunogenic peptides, which we used in this work. The efficacy of the DNA construct approach was reported in several studies (11, 12).

An important aspect is the migration and accumulation of tolDCs into disease-affected organs or lymph nodes. In order to enhance the therapeutic effect, different routes of administration (13) or manipulation of tolDCs through overexpression of chemokine receptors are used. The stability of tolDCs at the site of autoimmune reaction is also an important factor. Therefore, improved protocols to stabilize phenotype and functionality may be considered. One of the chemokines that can simultaneously influence migration and maintenance of the tolerogenic phenotype of DCs is CCR9. The chemokine receptor CCR9 is a G-protein coupled receptor that is present on various types of immune cells including DCs, CD4^+^ T cells, and B cells (14). CCR9 stimulates immune cells to move to gradients of its ligand CCL25, which is produced by intestinal and thymus epithelial cells (15). CCR9^+^DCs are known to be involved in the regulation of inflammation, food allergy, alloimmunity, and autoimmunity (16). The interaction of CCR9^+^DCs with lymphoid and myeloid cells in the thymus, secondary lymphoid tissues, and mucosal sites plays an important role in immune regulation (17). There is also evidence that increased expression of CCR9 on DCs leads to a decrease in MHC II and CD86 content by DCs, which may lead to a persistent tolerogenic DC phenotype (18).

In this study, we analyzed the efficacy of DCs that were transfected with DNA constructs encoding IL-10, type II collagen, and CCR9. The aim was to create DCs with a more robust tolerogenic phenotype capable of inducing T-reg, and thereby, activating immune tolerance mechanisms. We also planned to develop both nonspecific tolDCs using IL-10 and antigen-specific tolDCs using type II collagen for DC loading and induction of experimental arthritis. The use of the same antigen for induction and suppression of experimental arthritis allowed us to evaluate the efficacy of the approach based on the application of DNA constructs encoding regulatory molecules and peptides.

## Materials and methods

### Ethical statement

All manipulations with experimental animals were performed under isoflurane anesthesia according to the principles of humane treatment of animals. All protocols and methods of the study were approved by the Ethical Committee of NIIFKI, Novosibirsk, Russia (Protocol No. 119/10-10-2019).

### Laboratory animals

Male BALB/c mice aged 2–6 months were used in the study. Mice were kept in the vivarium of NIIFKI in the conditions of natural light and unrestricted access to food and water.

### Obtaining myeloid DCs in mice

Bone marrow cells (BMCs) were obtained by washing the medullary canal of femoral bones with phosphate-salt buffer (PBS). Then, the cells were washed twice: centrifuged for 10 minutes at 1500 rpm in the presence of PBS with the removal of supernatant. BMCs were cultured in 75 cm^3^ culture vials in the ratio of 1 million cells per 1 mL of the complete RPMI-1640 medium supplemented with 10% FCS (Biowest, France), 2 mM L-glutamine (Biolot, Russia), 10 mM HEPES (Biolot, Russia), 5×10-4M 2-mercaptoethanol (Sigma-Aldrich, USA), 80 μg/mL gentamicin (KRKA, Slovenia), and 100 μg/mL benzylpenicillin (Biolot, Russia). GM-CSF and IL-4 were used as growth factors at a concentration of 20 µg/mL. Half of the growth medium was changed every 2–3 days with the addition of ½ volume of growth factors. Cultures were collected on the 7th day for electroporation and cytometric analysis.

### Transfection

For further study of the obtained DCs after 7 days of culturing, the cell cultures were harvested and electroporated with DNA constructs encoding mouse chemotaxis receptor CCR9 (pmaxCCR9), encoding IL-10 production (pmaxIL-10), encoding type II collagen epitopes (pmaxCII), and non-coding control plasmid (p5). For electroporation, 5 × 10^6^ cells were resuspended in 50 μl OptiMEM (Thermo Fisher Scientific, USA) with or without the addition of 50 μg DNA plasmid and transferred to a 1 mm cuvette (BTX, USA). Electroporation was performed using a BTX 830 electroporator (BTX, USA) with a single pulse (260 V, pulse duration 200 μs). Electroporation was followed by a 10-minute resting phase. After the resting phase, cells were transferred for further cultivation in 5 mL a complete medium in a 6-well plate with the addition of GM-CSF and IL-4 at a concentration of 20 µg/mL. The following groups of transfected cells were created: Non-EP DCs – DCs without electroporation impact, DCp5 – DC culture, which was electroporated with a control non-coding plasmid p5, DCpIL10, DCpCII, DCpCCR9, DCpIL10+pCII, DCpCCR9+pCII+pIL10, DCpCCR9+pCII, and DCpCCR9+pCII.

### Evaluation of IL-10 production by transfected DCs

The pIL-10-transfected DCs were evaluated by enzyme immunoassay (EIA) for IL-10 concentration in the cell medium one day after transfection. EIA was performed using a commercial kit for IL-10 concentration determination. The procedure was performed according to the manufacturer’s instructions (Biolegend, USA), and the data were analyzed on a Varioskan LUX monochromator (USA).

### Co-culturing of DCs and lymphocytes

Splenocytes were isolated from the spleens of Balb/c mice and mixed with the obtained DCs at a ratio of 1:10. Five days after, the number of T-reg and the intracellular content of IL-10 and TGF-β in CD4^+^ T cells were evaluated by flow cytometry.

### Proliferative activity of CD4^+^-splenocytes in co-culture with T-reg in response to antigen

After 5 days of culturing of the obtained DCs and splenocytes, magnetic sorting of T-regs from the groups of DCpIL10 (nonspecific T-reg, hereafter referred to as non-AgT-reg) and DCpIL-10+CII+CCR9 (antigen-specific T-reg, hereafter referred to as AgT-reg) was performed. Magnetic sorting of CD4^+^CD25^+^ lymphocytes was performed according to the manufacturer’s protocol (MojoSort™ Mouse CD4^+^CD25^+^ Regulatory T Cell Isolation Kit, Biolegend, USA), using negative and then positive selection. The purity of the sorting was 98-99%. CD4^+^-splenocytes were obtained from mice with ACIA using the magnetic sorting method. CD4^+^ lymphocytes were then labeled with CFSE vital dye (Biolegend, USA) (5 mmol/ml) for 20 minutes according to the standard protocol and co-cultured with non-AgT-reg or AgT-reg in a ratio of 10:1 (2 × 10⁵ CD4^+^-splenocytes/2 × 10^4^ T-reg). After 48 hours, bovine Type II collagen (Collagen Type II, Bovine, Lyophilized, Sigma-Aldrich, Germany) was added at a concentration of 2 mg/ml of 25 µl per 2 × 10⁵ CD4^+^-splenocytes, and after another 3 days, the proliferative activity of CD4^+^-splenocytes was analyzed. A population of CD4^+^-splenocytes cultured with and without the addition of type II collagen was used as a control.

### Analysis of DC migration activity *in vitro*

To assess the migration activity of the obtained DCs *in vitro*, transwell systems with a pore size of 5 μm (Corning, USA) were used. A total of 500 μl of culture medium and various attractants were added to the lower section of the wells: 5 × 10⁵ Balb/c mouse thymus cells, 5 × 10⁵ Balb/c mouse lymph node cells, and as a control, culture medium without added attractants. 1 × 10⁵ of non-electroporated DCs (DC0 group) and CFSE prelabeled DCs of electroporated pmaxCCR9 (group DCpCCR9) of viable mice were added to the upper section. *In vitro* migration was assessed by fluorescence microscopy of the lower sections using an In Cell Analyzer 6000 with a Cell Culture Life Support Module (GE Healthcare, USA). Light-field and FITC (CFSE) channel microscopic images were taken every 4 hours up to 24 hours after cell seeding. For each well, 25 fields of vision in the transwell-membrane projection were analyzed. Migrated cells were counted using the In Cell Developer Toolbox 1.9.3 program (GE Healthcare, USA). The migration index for each group was calculated as [number of cells that migrated to the mentioned chemoattractants]/[number of cells that migrated to the culture medium].

### Analysis of T-reg content in co-culture of DCs and thymocytes

Thymocytes were isolated from the thymuses of Balb/c mice and mixed with the obtained DCs at a ratio of 1:5. Five days after, the number of T-reg was evaluated by flow cytometry.

### Flow cytometry

On day 7 of DC cultivation, the frequency of the obtained myeloid cells was analyzed by flow cytometry. A day after transfection, the phenotype of the obtained cells was evaluated by flow cytometry. For flow cytometry, 2 × 10^5^ transfected cells were harvested and incubated for 20 minutes in the dark at room temperature with the addition of fluorescent monoclonal antibodies. Anti-CD11c-PE-Cy7 and anti-SIRPα-PE-Cy5.5 were used to analyze the frequency of myeloid cells in the obtained culture; anti-CD11c-PE-Cy7, anti-CCR9-PE, anti-H-2Db-FITC, anti-CD80-PerCP, and anti-CD86-APC (Biolegend, USA) were used to analyze the phenotype. Using flow cytometry, the relative amount of FoxP3^+^CD25^+^CD4^+^CD3^+^ Tregs was studied in co-culture *in vitro*. Anti-FoxP3-PE, CD3 – BV421, CD4 – PerCP, and CD25 – FITC antibodies (Biolegend, USA) were used for flow cytometry. For intracellular FoxP3 staining, and for fixation and permeabilization, the True-Nuclear Transcription Factor Buffer Set (Biolegend, USA) was used according to the manufacturer’s instructions.

Flow cytometry was also used to study the ability of DCs transfected with DNA constructs encoding CCR9, IL-10, and type II collagen epitopes to induce FoxP3^hi^ Treg cells. For this purpose, anti-FoxP3-PE, CD3 – BV421, CD4 – PerCP, and CD25 – PE-Cy7 antibodies (Biolegend, USA) were used. For intracellular FoxP3 staining, and for fixation and permeabilization, the True-Nuclear Transcription Factor Buffer Set (Biolegend, USA) was used according to the manufacturer’s instructions.

To analyze the content of IL-10 and TGF-β, cells were first surface stained against CD3 – BV421 and CD4-PerCP (BioLegend, USA). After incubation with surface antibodies, the cells were washed in 500 μl of physiological saline with PBS. For intracellular analysis, after staining with surface antibodies, the cells were fixed in PBS containing 1% of paraformaldehyde, permeabilized with a 0.1% Tween-20 solution, and stained with monoclonal antibodies against IL-10-PE and TGF-β-APC.

Surface labeling with anti-CD4-PerCP (BioLegend, USA) was used to analyze the proliferative activity of CD4 in the co-culture of T-reg, CD4, and type II collagen.

### Antigen-collagen-induced arthritis protocol

The protocol of induction of antigen-collagen-induced arthritis (ACIA) was based on a model from the work of Baddack, Hartmann et al. (19).

BALB/c mice aged 2–6 months were used for ACIA induction. The first immunization was performed by subcutaneous injection of 100 μg of bovine serum methylated albumin (mBSA, Sigma-Aldrich, Germany) diluted in PBS and emulsified with the same volume of complete Freund’s adjuvant (CFA, Sigma-Aldrich, Germany). One week later, mice were re-immunized subcutaneously with a solution containing 100 μg of bovine collagen type II (Collagen Type II, Bovine, Lyophilized, Sigma-Aldrich, Germany) and 50 μg of mBSA emulsified with the same volume of Freund’s incomplete adjuvant (IFA, Sigma-Aldrich, Germany). In parallel with each immunization, 200 ng of Bordetella p. toxin (Pertussis toxin from Bordetella pertussis, PTX, lyophilized powder, Sigma-Aldrich, Germany) was injected intraperitoneally. Fourteen days after the last immunization, 50 μg of mBSA diluted in 20 μL of PBS was injected into the left knee joint cavity in the experimental group and 20 μL of PBS intra-articularly in the control group.

### *In vivo* study

According to the protocol, induction of a model of ACIA was performed.

Myeloid DCs were obtained by the method described above. On day 7, electroporation of exogenous DNA was performed using DNA constructs encoding the mouse chemotaxis receptor CCR9 (pmaxCCR9), the plasmid encoding IL-10 production (pmaxIL-10), and the plasmid encoding epitopes of collagen type II (pmaxCII). The following groups of transfected cells were created: DCpIL10, DCpIL10+pCII, DCpCCR9+pCII+pIL10, and DCpCCR9+pCII. DCs transfected with plasmid p5 were used as a control. The obtained DCs were collected on the next day after electroporation, washed twice with an RPMI-1640 medium, and diluted in a 0.9% saline solution at a concentration of 5 × 10⁵/150 μl. The obtained DC solution was injected into the tail vein of experimental animals on days 6–7 after induction of localized arthritis, when the first clinical signs appeared. The following treatment groups were created: 1 - DCpIL10, 2 - DCpIL10+pCII, 3 - DCpCCR9+pCII+pIL10, 4 - DCpCCR9+pCII, 5 - Control, saline solution was injected, 6 - Control, DCp5 was injected.

On days 7 and 14 after DC injection, visual analysis of paw swelling was performed using a caliper gauge, EIA of the content of antibodies to collagen type II in the serum of laboratory animals, and sampling of joints for histologic examination.

### Visual assessment of arthritis

Paw swelling was assessed by measuring the mean thickness of both hind paws with a 0–10 mm caliper gauge on days 0, 3, 6, and 7–23 after the induction of arthritis.

### Histological tests

On days 10 and 23 after the induction of arthritis, mice were killed by cervical dislocation. The hind paws were separated from the level of the knee joint. The obtained materials were fixed in a buffered 10% formalin solution for histological tests. Joint tissue sections were collected on slides, which were then deparaffinized and stained with hematoxylin and eosin. Inflammation was assessed as hyperplasia of the synovial membrane layer and leukocyte infiltration into the synovial membrane/joint space; tissue destruction was assessed by pannus formation and cartilage erosion.

### Enzyme immunoassay

The content of antibodies specific to murine collagen type II was measured in the serum of laboratory animals by enzyme immunoassay (EIA; murine IgG against murine collagen type II, Cayman Chemical, USA).

### Statistical analysis

Statistical analysis was performed using the Prism 8.0 program (GraphPad Software, USA). The significance of differences between samples was assessed using one-factor or two-factor ANOVA analysis of variance or the Mann-Whitney test. Data were presented as the median and interquartile range (25–75%). Differences in the compared parameters were considered statistically significant at p ≤ 0.05. Significant differences are illustrated in the figures by symbols and brackets.

## Results

### Analysis of the phenotype of transfected DCs and their ability to induce T-reg

**Figure 1:**
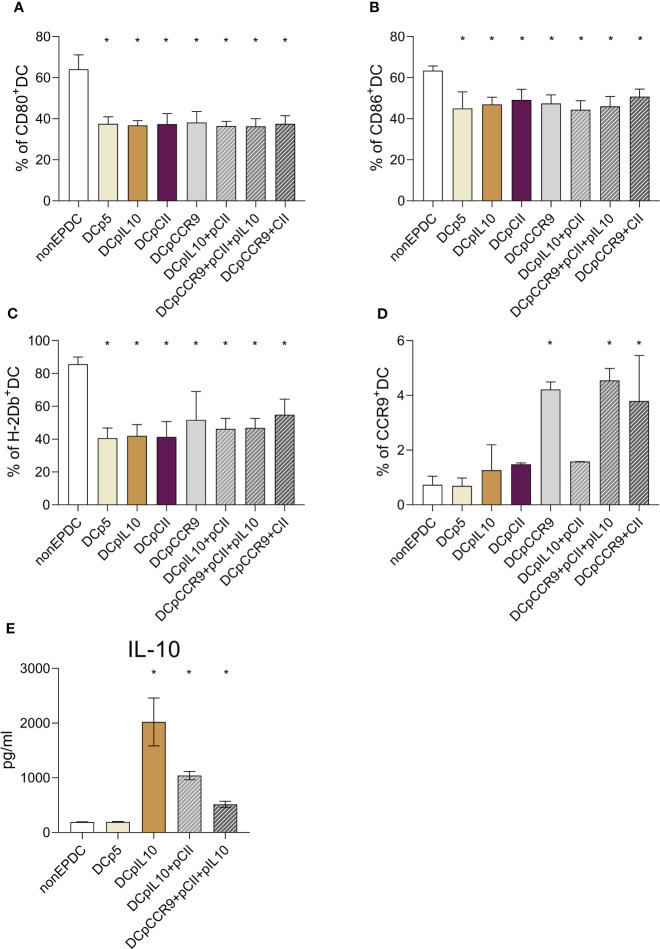
**(A-C)** Expression analysis of maturation markers of dendritic cells transfected with different plasmids: **(A)** relative content of DCs expressing CD80; **(B)** relative content of DCs expressing CD86; **(C)** relative content of DCs expressing H-2D^b^, (n = 8). * – statistically significant differences from the non-EP DC group (p ≤ 0.01). **(D)** CCR9 expression analysis of cultured DCs after transfection with different plasmids (n = 8). * – statistically significant differences from the group of non-EP DC, DCp5, DCpIL-10, DCCII and DCpIL10+pCII (p ≤ 0.001). **(E)** Analysis of the IL-10 content in the conditioned DC culture medium after transfection with different plasmids (n = 8). * – statistically significant differences from the group of non-EP DCs and DCp5 (p ≤ 0.001). Note: non-EP DC – DC culture that was not electroporated, DCp5 – DC culture electroporated with control non-coding plasmid (p5), DCpIL10 – DC culture electroporated with the experimental plasmid (pIL10), DCpCII – DC culture electroporated with the experimental plasmid (pCII), DCpCCR9 – DC culture electroporated with the experimental plasmid (pCCR9), DCpIL10+pCII – DC culture electroporated with the experimental plasmids (pIL10 and pCII), DCpCCR9+pCII+pIL10 – DC culture electroporated with the experimental plasmids (pCCR9, pCII and pIL10), DCpCCR9+pCII – DC culture electroporated with the experimental plasmids (pCCR9 and pCII).

We evaluated the phenotype of transfected and non-transfected DCs (**Figures 1A–C**). The analysis of the obtained DCs phenotype revealed a significant decrease in the expression of CD80, CD86, and H-2Db by the obtained cells compared to the DCs that were not transfected. This finding may indicate the formation of a tolerogenic phenotype of transfected DCs. All DC transfected, even with an empty p5 plasmid, had an immature phenotype, compared with DC without transfection, which turned out to be mature. Perhaps such a difference in data is related to the very method of delivery of exogenous DNA - electroporation transfection, which in itself can lead to the preservation of an immature DC phenotype in our protocol.

Next, we evaluated the CCR9 expression of the investigated myeloid DCs (**Figure 1D**). It was revealed that the groups of DCs where pmaxCCR9 was used for transfection showed an increased expression of the chemotaxis receptor compared to other groups.

To assess the efficiency of transfection, we studied the level of IL-10 production by DCs a day after transfection (**Figure 1E**). A significant increase in cytokine content was shown in the conditioned media of all DC groups that were transfected with the pIL-10 plasmid.

Thus, transfection was successful because CCR9 expression and IL-10 production by dendritic cells increased.

**Figure 2:**
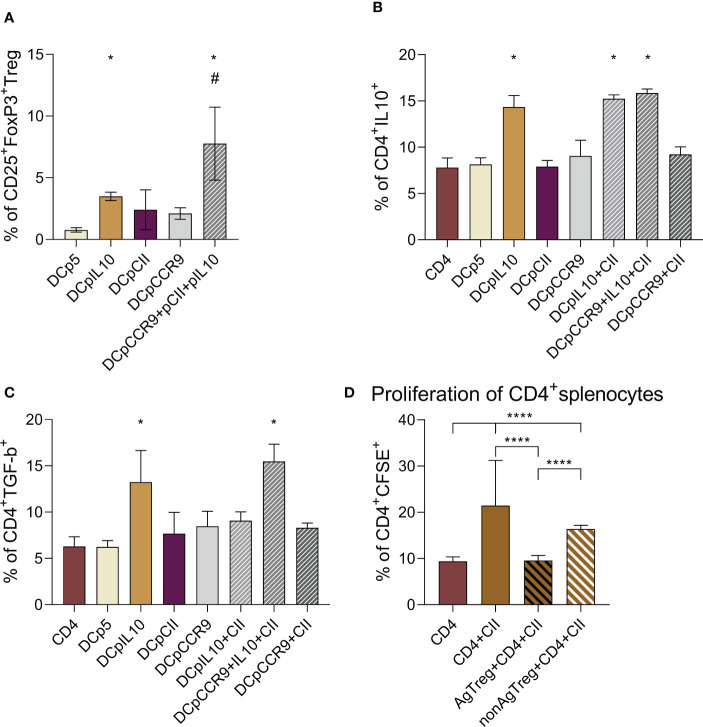
**(A)** Analysis of the relative content of T-reg (CD25^+^FoxP3^+^) in the co-culture of the studied DCs and splenocytes, (n = 8). * – statistically significant differences groups DCpIL-10 and DCpIL-10+pCII+pCCR9 from the DCp5 group, # – statistically significant differences of DCpIL10+pCII+pCCR9 from all other groups (one-way ANOVA, p ≤ 0.0001). Analysis of the intracellular IL-10 **(B)** and TGF-β **(C)** content in DC and CD4^+^splenocytes co-culture, (n = 8). * – statistically significant differences of the DCpIL-10, DCpIL-10+pCII, and DCpIL-10+pCII+pCCR9 groups from the other groups (one-way ANOVA, p ≤ 0.0001). **(D)** Evaluation of the proliferative activity of CD4^+^splenocytes in co-culture with T-reg upon the addition of type II collagen, (n = 6). CD4 alone – CD4^+^splenocytes without addition of T-reg and type II collagen, CD4+CII – CD4^+^splenocytes with addition of type II collagen, AgT-reg+CD4+CII – antigen-specific T-reg, CD4^+^splenocytes and type II collagen, non-AgT-reg +CD4+CII – nonspecific T-reg, CD4^+^splenocytes and type II collagen. Brackets indicate statistically significant differences (one-way ANOVA, **** - P < 0.0001).

Then, we obtained co-culture of DCs and splenocytes and analyzed the number of T-reg on day 5. There was a significantly increased T-reg content in DCpIL-10, DCpIL-10+pCII+pCCR9, and DCpCCR9+pCII groups compared to other groups (**Figure 2A**).

To show that tolDCs in co-culture induce CD4^+^ T cells to produce the immunosuppressive cytokines IL-10 and TGF-β, we analyzed the intracellular content of IL-10 (**Figure 2B**) and TGF-β (**Figure 2C**) in CD4^+^ T cells. It was shown that in the co-culture of DCs and CD4^+^ cells, the intracellular content of IL-10 significantly increased in DCpIL-10, DCpIL-10+pCII, and DCpIL-10+pCII+pCCR9, and TGF-β significantly increased in the DCpIL-10 and DCpIL-10+pCII+pCCR9 groups.

Thus, we obtained an increase in relative T-reg and intracellular IL-10 and TGF-β content in target groups of transfected DCs in co-culture with splenocytes.

### Evaluation of the proliferative activity of CD4^+^ splenocytes in response to antigen

To analyze the proliferative activity of CD4^+^ splenocytes (obtained from mice with ACIA) in a combined culture with antigen-specific (obtained from the DCpCCR9+pCII+pIL-10 group) and non-specific T-regs (obtained from the DCpIL-10 group), cells were stimulated with type II collagen (**Figure 2D**). Type II collagen significantly increased the proliferation of CD4^+^ splenocytes that was inhibited in the presence of AgT-regs and non-AgT-regs. In particular, Ag-T-regs completely abrogated splenocyte proliferation, whereas non-AgT-regs decreased it to a lesser extent.

The data obtained indicate that antigen-specific T-regs are able to efficiently suppress the type II collagen-induced proliferation of CD4^+^ splenocytes, thus might support the development of immune tolerance.

### *In vitro* analysis of the migration capabilities of DCs transfected with CCR9 DNA constructs and analysis of T-reg content in co-culture of DCs and thymocytes

The next step was to study the migration activity of DCs transfected with a DNA construct encoding CCR9 to thymus cells and lymph node cells *in vitro* using transwell systems. The migration index was calculated as the ratio of the number of cells that migrated to thymus cells or lymph node cells to the number of cells that migrated to the culture medium without chemoattractants.

**Figure 3:**
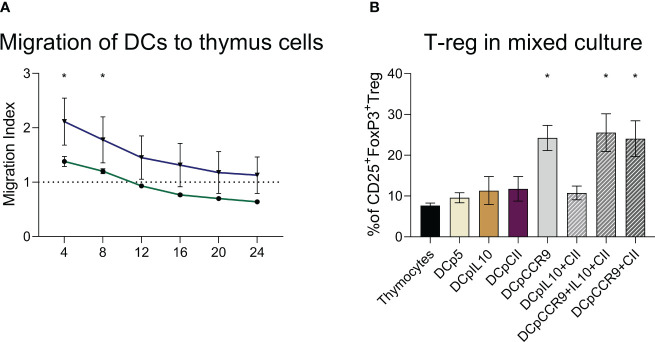
**(A)** Evaluation of dendritic cell migration ability, (n = 6). DCs are dendritic cells unaffected by electroporation, DCpCCR9 are DCs transfected with the CCR9 plasmid. Medians and interquartile range. * – statistically significant differences from the DC group (two-way ANOVA, P < 0.0001). **(B)** Analysis of T-reg content in co-culture of DCs and thymocytes, (n = 6). * – statistically significant differences of the DCpCCR9, DCpCCR9+pCII, and DCpIL-10+pCII+pCCR9 groups from the control groups Thymocytes and DCp5 (p ≤ 0.0001).

It was found that 4 and 8 hours after seeding, DCs transfected with pmaxCCR9 were 1.5 times more active in migrating to thymus cells compared to non-electroporated DCs (**Figure 3A**).

On day 5 of the co-culturing of the obtained DCs and thymocytes, the content of T-reg was analyzed. The experiment showed that in the groups where the DNA construct encoding CCR9 was used for transfection, there was a significant increase in the content of T-reg compared to the other groups (**Figure 3B**).

As this study showed, transfection by the DNA construct encoding CCR9 leads to the fact that DCs acquire a higher migration potential in relation to thymic cells than nontransfected DCs. CCR9^+^DC also contribute to the induction of T-reg cells when co-cultured with thymocytes.

### Evaluation of the impact of the obtained DCs on the course of modeled arthritis in mice

**Figure 4:**
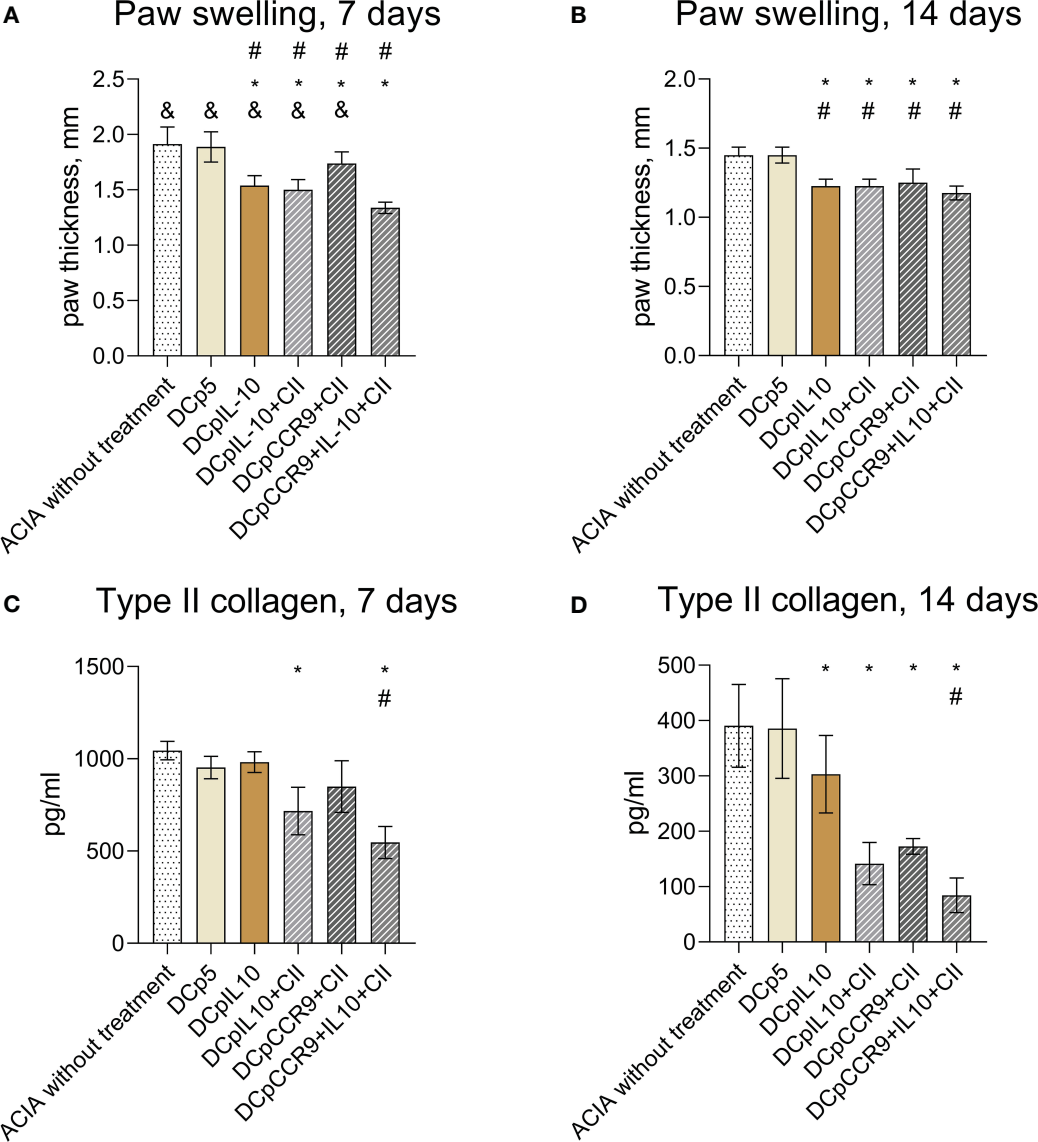
Comparison of paw swellings of laboratory animals on days 7 **(A)** and 14 **(B)** after the initiation of therapy (n = 8–12). ACIA without treatment and DCp5 are the control groups. Medians and interquartile range. # - statistically significant differences from the control group ACIA without treatment, * - statistically significant differences from the control group DCp5, & - statistically significant differences from the DCpCCR9+pCII+pIL10 group (one-way ANOVA, P < 0.0001). Comparison of antibody levels to type II collagen in laboratory animals on days 7 **(C)** and 14 **(D)** after treatment (n = 8–12). ACIA without treatment and DCp5 are the control groups. Medians and interquartile range. * – statistically significant differences from the control groups, # – statistically significant differences the DCpCCR9+pCII+pIL10 group (one-way ANOVA, P < 0.001).

After the injection of the obtained DCs to mice with ACIA, we compared paw swelling and levels of antibodies to type II collagen on days 7 (**Figure 4A**) and 14 of treatment (**Figure 4B**). It was shown that paw swelling in all experimental groups was significantly less than in control groups both on days 7 and 14. Also, paw swelling of the DCpCCR9+pCII+pIL10 group was significantly less pronounced than in all groups on day 7.

The analysis of the levels of antibodies to type II collagen (**Figures 4C, D**) showed a significant decrease in the antibody content only in the groups DCpIL10+pCII and DCpCCR9+pCII+pIL10, compared with the control group on day 7 of treatment. On day 14 after the beginning of treatment in all experimental groups, except DCp5, a decrease in the titer of antibodies to collagen type II was detected. Moreover, the DCpCCR9+pCII+pIL10 group showed the lowest level of antibodies to type II collagen compared to the other groups both on days 7 and 14.

**Figure 5:**
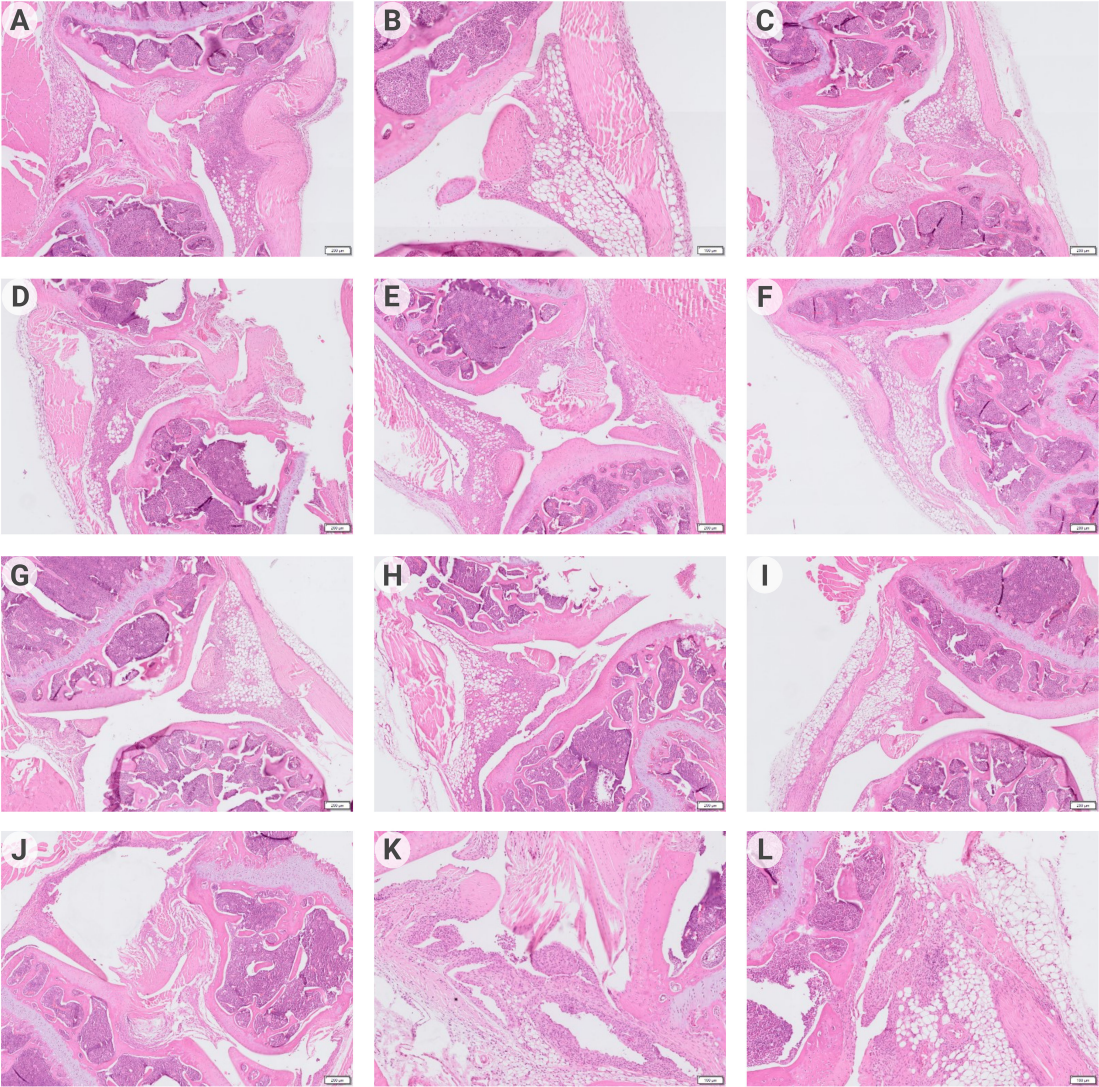
Data of histologic analysis of mice joint tissues (n = 4–6). On day 7 after treatment: group 1 – pronounced lymphocytic infiltration of the synovial stroma, formation of a subchondral cyst **(A)**; group 2 – pannus formation, weak inflammatory infiltration of the synovial membrane **(B)**; group 3 – thickening of the synovial membrane with weak lymphocytic infiltration, formation of synovial villi **(C)**; group 4 – moderate infiltration of the synovial membrane, penetration of the synovial membrane into the articular cartilage **(D)**; group 5 – synovial membrane ingrowth into the bone tissue near the articular cartilage, moderate lymphocytic infiltration of the synovial membrane with its thickening and formation of villi **(E)**; group 6 – weak inflammatory infiltration of synovia, pannus is visualized **(F)**. On day 14 after treatment: group1 – infiltration of the synovial membrane by lymphocytes **(G)**; group 2 – moderate infiltration of the synovial membrane of the joint, proliferation of fibroblasts **(H)**; group 3 – no visible pathologic changes were detected **(I)**; group 4 – moderate lymphocytic infiltration of the subsynovial stroma **(J)**; group 5 – thickening and numerous outgrowths (villi) of the synovial membrane, small piles of leukocytes in the joint cavity **(K)**; group 6 – synovial membrane overgrowth with pannus formation, lymphocytic infiltration of the subsynovial stroma **(L)**.

The analysis of the histological specimens (**Figures 5A–F**) obtained one week after the start of treatment showed that all animals had inflammatory infiltration of the synovial membrane. In some animals, the formation of pannus, penetration of synovium into articular cartilage, and thickening of the synovial membrane were revealed. However, 14 days after DC injection (**Figures 5G–L**), the most manifested changes were found only in the control groups – thickening and infiltration of the synovial membrane with lymphocytes; in one animal, with the formation of villi and small groups of leukocytes in the joint cavity. In the DCpCCR9+pCII+pIL10 group, pathologic changes were absent in all animals. In the DCpIL10 group, two animals had synovial membrane villi formation and weak lymphocytic infiltration. The DCpIL10+pCII and DCpCCR9+pCII groups had similar pathomorphologic phenomena – moderate lymphocytic infiltration. Consequently, minimal changes were detected in the DCpIL10 group on day 14 of treatment, and the absence of pathology in animals of the DCpCCR9+pCII+pIL10 group on day 14 of treatment. The most pronounced changes were observed in the control groups.

Thus, i/v injection of the studied DCs resulted in decreased levels of paw swelling and type II collagen antibodies in the experimental groups.

## Discussion

In this study, the ability of nonspecific and antigen-specific DCs to induce immune tolerance through T-reg formation in modeled arthritis was investigated. DCs were also transfected with the chemokine CCR9 and IL-10 to enhance the stability of the tolerogenic DC phenotype, and to engage not only peripheral immunologic tolerance mechanisms but also central ones, as CCR9 leads to DC migration to the thymus. Type II collagen was chosen as an antigen.

The resulting DCs had an immature or tolerogenic phenotype (**Figures 1A–C**), which is required for T-reg induction. Immature DCs induce tolerance by providing insufficient stimulatory signals for T cells. In steady-state conditions, tolDCs capture autoantigens from apoptotic cells, commensal bacteria, and antigens (20, 21). They then migrate to draining lymph nodes without sufficient maturation, resulting in the induction of tolerance. The tolerance-inducing properties of tolDCs are determined by their origin and a number of factors such as cytokines, growth factors, and transcriptional programming, which together confer suppressive functions to these cells (20–22). Mature DCs are activated and switch to a stimulatory phenotype in the presence of maturation signals, which subsequently leads to the induction of effector/cytotoxic T-cell responses (23).

IL-10 and CCR9 were used to fix the stable tolerogenic phenotype of DCs. DCs producing high levels of IL-10 were obtained (**Figure 1E**). IL-10^+^DCs are known to stimulate CD4^+^IL-10-cell formation *in vitro* and *in vivo* – these cells are able to suppress T-cell proliferation and trigger FoxP3^+^T-reg cells (24–26). There is evidence that tolDCs producing high levels of IL-10 express ILT4 and HLA-G and are able to induce Tr1 cells. The interaction of ILT4/HLA-G molecules on DCs increases IL-10 production by these cells, which in turn, stimulates ILT4 and HLA-G expression on other DCs (25).

Currently, little is known about the presence or functional effects of CCR9 on myeloid DCs. However, there is evidence that DCs expressing high levels of CCR9 are less mature than DCs expressing low levels of CCR9 (27). This occurs because increased expression of CCR9 on DCs leads to decreased MHC II and CD86 content on DCs (18). CCR9 may also prevent DC maturation by inhibiting the V-ATPase domain assembly or activating the NFκB pathway to regulate TSLP secretion (28, 29). TSLP is a molecule known to be secreted by DCs and promote the induction of T-reg (30). According to our data, CCR9^+^DCs had significantly reduced levels of DC maturation markers (**Figures 1A–C**). It is worth noting that all transfected DCs, even with an empty p5 plasmid, had an immature phenotype compared to DCs without transfection, which turned out to be mature. It is possible that this difference in data is associated with the very method of exogenous DNA delivery – transfection by electroporation, which itself may lead to the preservation of the immature DC phenotype in the protocol. There is evidence that in addition to its effect on DCs, CCR9 can influence the differentiation of regulatory Foxp3^+^CD4^+^T-cells and affect IL-2 production through internal signaling in DCs (17).

It should be noted myeloid DCs were used, which, after transfection with a DNA construct encoding CCR9, showed the highest migration potential to thymic cells compared to untransfected DCs (**Figure 3A**). Next, it was decided to test how the cells would behave in culturing conditions with thymic cells. It was shown that CCR9^+^ DCs promoted the production of T-reg cells (**Figure 3В**). It is also known that myeloid DCs are cells expressing CCR7, which promotes DC migration to lymph nodes. Given the described data, one can assume that not only peripheral immune tolerance mechanisms are affected but also central ones.

TGF-β and IL-10 are the most studied molecules that are involved in T-reg generation (31). This study showed that in co-cultured DCs and CD4^+^splenocytes the intracellular IL-10 content significantly increased in the DCpIL-10, DCpIL-10+pCII, and DCpIL-10+pCII+pCCR9 groups; and TGF-β significantly increased in the DCpIL-10 and DCpIL-10+pCII+pCCR9 groups, i.e. in both non-specific DCs and antigen-specific DCs. Interestingly, a significantly increased T-reg content was obtained in the same groups: DCpIL-10 and DCpIL-10+pCII+pCCR9 compared to the other groups. Analyzing the proliferation level of CD4^+^lymphocytes in response to autoantigen (**Figure 2D**), the proliferation level was decreased in the nonspecific T-reg group compared to the control. In the antigen-specific T-reg group, suppression of the proliferative activity of CD4^+^ lymphocytes was revealed, with a significantly lower level compared with the non-AgT-reg, and equal to the control without collagen addition. Thus, one can suggest the emergence of antigen-specific tolerance in this experiment.

Mouse models of arthritis are indispensable tools for developing new methods of therapy. Existing models do not accurately recreate RA or are difficult to reproduce. Most models are reproduced in mice with a specialized genetic background (32). The best-known model of experimental arthritis in mice is collagen-induced arthritis (CIA) (33). In this model, arthritis develops in approximately 80% of mice, but only in strains carrying MHC class II subtypes IAq, IAr, and H-2q, and has a remitting disease course (33). According to the literature, the combined model induced by methylated bovine serum albumin (mBSA) and collagen most closely mimics the pathogenesis of RA in Balb/c mice (19). The Balb/c line was shown to be most susceptible to antigen-collagen-induced arthritis (AIA/CIA) (19). In the classical model of CIA, mice of the C57BL/6 line are used. Due to genetic differences between Balb/c and C57BL/6 mice, such as different MHC haplotypes, these lines respond differently in models of infectious processes and AIDs (34). While C57BL/6 mice develop unexpressed arthritis in AIA/CIA models, Balb/c mice show a rapid onset of inflammation and antibody formation to the cyclic citrullinated peptide (19). The AIA/CIA model is one of the closest in pathogenesis to RA. In our previous work, we described the development of ACIA in mice and showed the immunologic mechanisms and validity of the use of this model in research activities and for testing new therapeutic agents for RA (35).

Administration of tolDCs transfected with DNA constructs encoding epitopes of type II collagen, IL-10, and CCR9 to laboratory animals with ACIA allowed reducing the severity of paw swelling in experimental mice (**Figures 4A, B**) and decrease the level of antibodies to type II collagen (**Figures 4C, D**) and pathologic changes in joint tissues (**Figure 5**). Moreover, the DCpCCR9+pCII+pIL10 group showed the lowest levels of the compared parameters and the absence of pathologic changes in a histologic examination on day 14 after the treatment administration. It can be assumed that antigen-specific suppression was observed, since the DCpCCR9+pCII+pIL10 group showed the lowest level of antibodies to type II collagen compared to the other groups, both on days 7 and 14.

## Conclusion

To conclude, DCs transfected with DNA constructs encoding epitopes of type II collagen, IL-10, and CCR9 promote antigen-specific tolerance, control inflammation, and reduce the severity of experimental arthritis through the induction of T regulatory cells, IL-10, and TGF- β.

Thus, it is possible to modulate immune reactions and treat AIDs using DCs transfected with DNA constructs. The developed method can be effective in clinical practice. However, further studies are needed to determine the target antigens of therapy, determine the dosage, duration and frequency of administration, as well as maintain the positive effect of therapy over time.

## Data availability statement

The original contributions presented in the study are included in the article/supplementary material. Further inquiries can be directed to the corresponding author.

## Ethics statement

The animal study was approved by Local Ethics Committee of Research Institute of Fundamental and Clinical Immunology. The study was conducted in accordance with the local legislation and institutional requirements.

## Author contributions

MF: Conceptualization, Data curation, Investigation, Methodology, Project administration, Writing – original draft, Writing – review & editing. VK: Conceptualization, Investigation, Methodology, Writing – review & editing. AB: Investigation, Writing – review & editing. JS: Methodology, Writing – review & editing. JP: Data curation, Investigation, Writing – review & editing. OT: Data curation, Formal analysis, Writing – review & editing. EI: Data curation, Formal analysis, Writing – review & editing. AM: Methodology, Resources, Writing – review & editing. SS: Conceptualization, Writing – review & editing.
